# Clinical significance of long non-coding RNA DUXAP8 and its protein coding genes in hepatocellular carcinoma

**DOI:** 10.7150/jca.47902

**Published:** 2020-08-25

**Authors:** Xiang-Kun Wang, Xi-Wen Liao, Rui Huang, Jian-Lu Huang, Zi-Jun Chen, Xin Zhou, Cheng-Kun Yang, Chuang-Ye Han, Guang-Zhi Zhu, Tao Peng

**Affiliations:** 1 Department of Hepatobiliary Surgery, The First Affiliated Hospital of Guangxi Medical University, Nanning, 530021, Guangxi Province, China;; 2 Department of Hematology, The First Affiliated Hospital of Guangxi Medical University, Nanning, 530021, Guangxi Province, People's Republic of China;; 3 Department of Hepatobiliary Surgery, The Third Affiliated Hospital of Guangxi Medical University, Nanning 530031, Guangxi Province, China.

**Keywords:** long non-coding RNA, DUXAP8, protein-coding gene, hepatocellular carcinoma, molecular mechanism

## Abstract

**Backgrounds:** Hepatocellular carcinoma (HCC) is a lethal malignancy worldwide that is difficult to diagnose during the early stages and its tumors are recurrent. Long non-coding RNAs (lncRNAs) have increasingly been associated with tumor biomarkers for diagnosis and prognosis. This study attempts to explore the potential clinical significance of lncRNA DUXAP8 and its co-expression related protein coding genes (PCGs) for HCC.

**Method:** Data from a total of 370 HCC patients from The Cancer Genome Atlas were utilized for the analysis. DUXAP8 and its top 10 PCGs were explored for their diagnostic and prognostic implications for HCC. A risk score model and nomogram were constructed for prognosis prediction using prognosis-related genes and DUXAP8. Molecular mechanisms of DUXAP8 and its PCGs involved in HCC initiation and progression were investigated. Then, potential target drugs were identified using genome-wide DUXAP8-related differentially expressed genes in a Connectivity Map database.

**Results:** The top 10 PCGs were identified as:* RNF2*, *MAGEA1*, *GABRA3*, *MKRN3*, *FAM133A*, *MAGEA3*, *CNTNAP4*, *MAGEA6*, MALRD1, and *DGKI*. Diagnostic analysis indicated that DUXAP8, *MEGEA1*, *MKRN3*, and *DGKI* show diagnostic implications (all area under curves ≥0.7, p≤0.05). Prognostic analysis indicated that DUXAP8 and *RNF2* had prognostic implications for HCC (adjusted p=0.014 and 0.008, respectively). The risk score model and nomogram showed an advantage for prognosis prediction. A total of 3 target drugs were determined: cinchonine, bumetanide and amiprilose and they may serve as potential therapeutic targets for HCC.

**Conclusion:** Functioning as an oncogene, DUXAP8 is overexpressed in tumor tissue and may serve as both a diagnostic and prognosis biomarker for HCC. *MEGEA1*, *MKRN3*, and *DGKI* maybe potential diagnostic biomarkers and *DGKI* may also be potentially prognostic biomarkers for HCC.

## Introduction

By 2018, it was estimated that nearly 841,800 new liver cancer cases and 781,631 deaths due to liver cancers were recorded across 20 world regions 1. Hepatocellular carcinoma (HCC) accounts for approximately 80% of primary liver cancers, and ranks fifth among the most common malignancies and the third leading cause of cancer-related mortality worldwide 2, 3. Hepatitis B or C (HBV) viral infections are known to cause chronic liver cirrhosis and account for approximately 80-90% of all the HCC cases 4. Other risk factors include excessive alcohol consumption, obesity, aflatoxin B contamination, iron overload, and environmental pollutants 5, 6. HCC is a fatal malignancy characterized by high metastasis and recurrence after surgery leading to poor prognosis and very low survival rates 7, 8. Several treatments including hepatic resection, liver transplantation, image-guided tumor ablation, transcatheter tumor therapy, and systemic therapy have been developed to control HCC 9. However, patient survival rates are still low despite these treatments. The 5-year survival rate of HCC is about 7% 10. Identification of novel diagnostic biomarkers and therapeutic targets is crucial for improving prognosis of HCC patients.

Long non-coding RNAs (lncRNAs) regulate normal cellular functions 11, 12. They also participate in various biological and pathological processes such as tumorigenesis 13. Overexpression of CCAT2 suppresses cell migration, invasion, and growth, and induce early apoptosis of glioma cells 14. LncRNAs have been reported to regulate pathogenesis of HCC 15. For instance, HULC 16 and LINC00974 17 have been reported to participate in HCC development and progression. LINC00673 regulates HCC carcinogenesis via microRNA-205 and is upregulated in HCC tissues and cell lines 12.

DUXAP8, a pseudogene derived from lncRNA, promotes growth of pancreatic carcinoma cells by epigenetically silencing CDKN1A and KLF2 18. It has also been found to enhance renal cell carcinoma by downregulating microRNA-126 19. DUXAP8 is differentially expressed in bladder cancer, its downregulation inhibits cell invasion and proliferation and leads to cell apoptosis 20. Lijuan *et al.* reported that DUXAP8 expression was positively correlated with lymph node metastasis and clinical stage in esophageal cancer 21. This suggested that DUXAP8 is up-regulated and increases metastasis of renal cell carcinoma cells 22. DUXAP8 knockdown resulted in clear cell cycle arrest at the G0/G1 phase 23. This showed that DUXAP8 may be an effective prognostic biomarker and treatment target in non-small cell lung cancer 23. Genome-wide analysis showed that DUXAP8 was not only differentially expressed in esophageal cancer but was a diagnostic and therapeutic target for cancer 24. Despite these studies, little is known about the association between DUXAP8 and HCC. Therefore, we assessed the diagnostic and prognostic implications, molecular mechanism and potential target drugs of DUXAP8 in HCC.

## Materials and Methods

### Data processing and identification of protein-coding gene (PCG)

HCC patient expression data was downloaded from The Cancer Genome Atlas database (TCGA, https://cancergenome.nih.gov/). Criteria of patient selection as following: patients were pathologically diagnosed primary HCC, vital data including HBV infection status, alcohol history, vascular invasion and radical resection should be collected, all the patients have definite alive/die status and survival time. LncRNAs function together with their co-expression-related PCGs and co-expression coefficient. Therefore, evaluated to acquire its PCGs was conducted using R 3.5.0 (https://www.r-project.org/) 25, 26. The top 10 PCGs and DUXAP8 underwent further analysis. In addition, low and high expressions of DUXAP8 and the top 10 PCGs were used as cut-off median expressions of DUXAP8.

### Body distribution, tumor, and non-tumor expression

Body distribution of DUXAP8 and its top 10 PCGs were obtained from the Gene Expression Profiling Interactive Analysis database (GEPIA, http://gepia.cancer-pku.cn/index.html) 27. Scatter plots of the tumor and non-tumor tissues were plotted using Graphpad 7.0.

### Determination of diagnostic and prognostic significance of DUXAP8 and its PCGs

Diagnostic Receiver Operating Characteristic (ROC) curves of DUXAP8 and the PCGs were depicted using data from tumor and non-tumor tissue expressions using Graphpad 7.0. An Area Under Curve (AUC) value ≥ 0.7 represented high diagnostic significance. The joint-effect analysis was performed using the diagnosis-related lncRNA and genes.

In addition, prognostic significance was calculated and visualized using Kaplan-Meier plots. Multivariate analysis was performed using prognosis-related clinical factors. The joint-effect analysis was also performed using prognosis-related lncRNA and genes.

### Construction of the risk score model and nomogram

A risk score model and nomogram were constructed for HCC prognosis prediction. Risk scores, patient survival status, DUXAP8 gene expression heat maps, Kaplan-Meier plot, and time-dependent ROC curves were entered into the model. The risk score was formulated as follows: risk score = expression of gene_1_ x β_1_ of gene_1_ + expression of gene_2_ x β_2_ of gene_2_ +… + expression of gene_n_ x β_n_ of gene_n_ 28, 29. Contribution coefficients (β) were obtained from the multivariate Cox proportional hazards regression model.

The nomogram was constructed using prognosis-related genes, DUXAP8, and clinical factors. Differential expressions and clinical factors indicate varied points. The 1, 3-, and 5- year overall survivals (OS) were included in the nomogram. OS prediction at 1, 3, and 5 year can be found accordingly from the points.

### Gene set enrichment analysis (GSEA)

GSEA was performed to assess molecular mechanisms of DUXAP8 and the PCGs. Gene ontologies (GOs), including biological processes (BP, c5.bp.v6.1.symbols.gmt,), cellular components (CC, c5.cc.v6.1.symbols.gmt), molecular functions (MF, c5.mf.v6.1.symbols.gmt), and Kyoto Encyclopedia of Genes and Genomes (KEGG, c2.cp.kegg.v6.1.symbols.gmt) pathway sets were used to find the biological processes associated with DUXAP8 and its PCGs.

### Co-expression matrix, gene-gene interaction (GGI) and protein-protein interaction (PPI) network

The co-expression matrix for DUXAP8 and its PCGs of Pearson correlation were constructed using R 3.5.0. Different colors indicate either positive or negative correlations. The GGI network was constructed and their co-expression relationship with other genes was determined using the geneMANIA plugin of Cytoscape software 30, 31. The PPI network was constructed using the STRING database (https://string-db.org/) 32. Co-expression and co-occurrence interactions are presented using STRING.

### Pharmacological target drug identification and enrichment analysis

Genome-wide differentially expressed genes (DEGs) in volcano plots and heatmaps were determined and plotted using the edgeR method 33. Results with a ∣Fold change∣ of ≥ 2 and a p-value of ≤0.5 were used for further analysis. Target drugs were then acquired using these DEGs obtained from the Connectivity Map database (https://portals.broadinstitute.org/cmap/). Positively correlated drugs were considered as potential targets. The 2D and 3D structure of the target drugs were obtained from PubChem Compound (https://www.ncbi.nlm.nih.gov/pccompound/).

Enriched metabolic pathways and biological processes were analyzed using the Database for Annotation, Visualization and Integrated Discovery (DAVID, https://david.ncifcrf.gov/) 34, 35. Visualization of GO was performed using the BinGO plugin of Cytoscape software 36.

### Validations of PCGs in public databases

For further confirming potential values of these PCGs in HCC, we further used HCCDB 37 (http://lifeome.net/database/hccdb/home.html) and oncomine 38, 39 (https://www.oncomine.org/resource/main.html) databases to confirm our results.

### Statistical analysis

Survival and statistical analyses were performed using SPSS 16.0 (IBM, Chicago, IL). Median survival time, log-rank p-value, adjusted p-value, 95% confidence interval (CI) and hazard ratio (HR) were calculated using the Kaplan-Meier method and Cox proportional hazards regression model. A p-value of ≤ 0.05 was statistically significant.

## Results

### Demographic characteristics and co-expression-related PCGs of DUXAP8

A total of 370 HCC patients were enrolled in this study. Clinical factors found to be linked with HCC were HBV infection, radical resection, and tumor stage OS (p<0.0001, =0.007, and <0.0001, respectively). Detailed demographic characteristics are shown in **Supplementary Table 1**. Co-expression-related PCGs were identified and are shown in **Supplementary Table 2**. The top 10 PCGs were; *RNF2*, *MAGEA1*, *GABRA3*, *MKRN3*, *FAM133A*, *MAGEA3*, *CNTNAP4*, *MAGEA6*, MALRD1, and *DGKI* (**Table 1**). Pearson correlation coefficient R was used to evaluate the correlation between these PCGs and DUXAP8. PCGs with an ∣R∣ value of ≥0.2 were visualized in the network (**Figure 7B**).

### Body maps, expression of DUXAP8 and the top 10 PCGs

**Figure 1:**
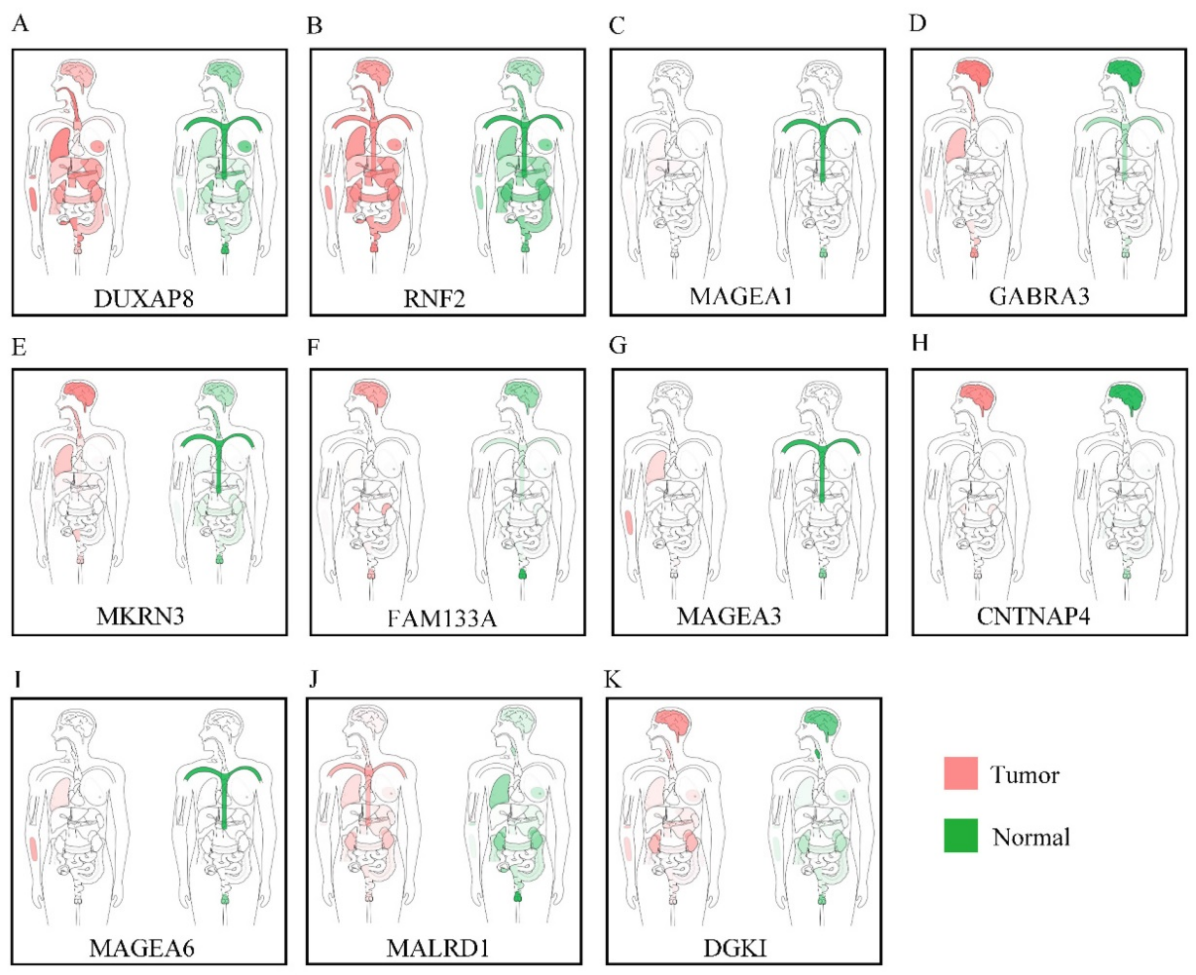
Body map of the expressions of DUXAP8 and its co-expression-related protein-coding genes. A-K: Body map of the expressions of DUXAP8, *RNF2*, *MAGEA1*, *GABRA3*, *MKRN3*, *FAM133A*, *MAGEA3*, *CNTNAP4*, *MAGEA6*, *MALRD1*, and *DGKI*, respectively.

**Figure 2:**
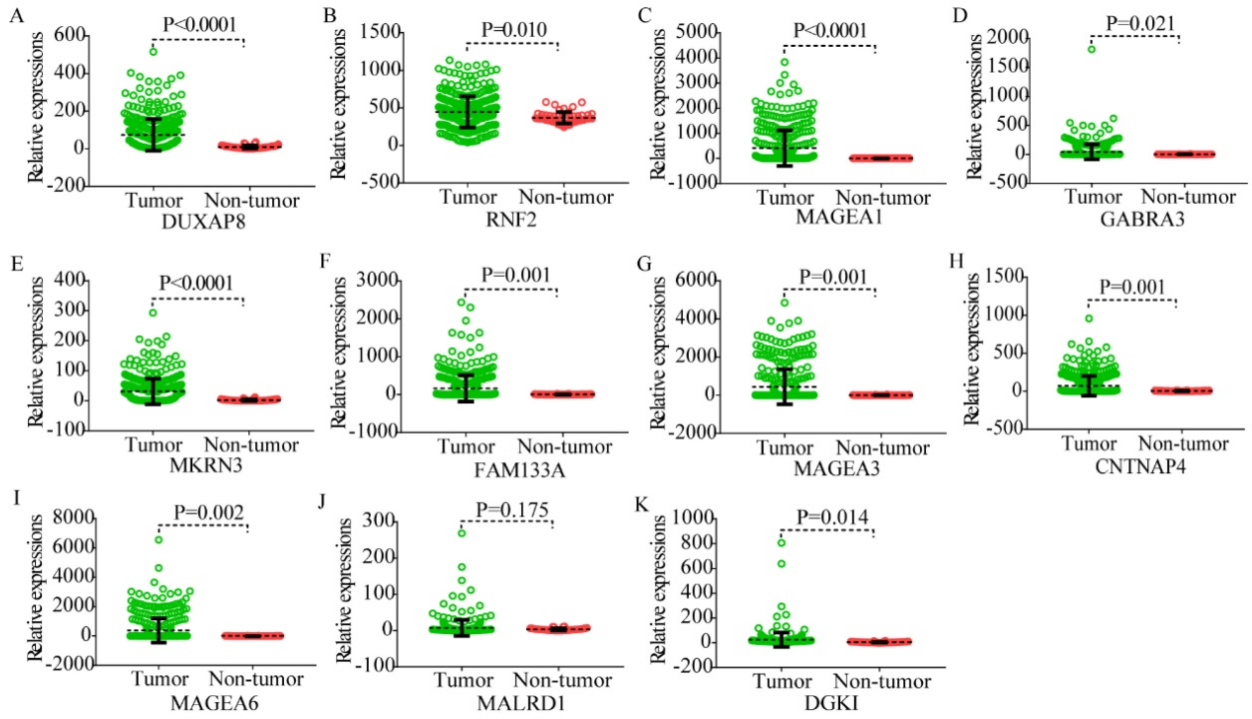
Scatter plots of DUXAP8 and its co-expression-related protein-coding genes in tumor and non-tumor tissues. A-K: Scatter plots of DUXAP8, *RNF2*, *MAGEA1*, *GABRA3*, *MKRN3*, *FAM133A*, *MAGEA3*, *CNTNAP4*, *MAGEA6*, *MALRD1*, and *DGKI*, respectively.

Body maps of DUXAP8 and the top 10 PCGs showed that DUXAP8 and *RNF2* were highly expressed in liver in comparison to other PCGs (**Figure 1**). In comparison, DUXAP8 and *RNF2* were highly expressed in kidney, lungs, and intestines relative to other PCGs. Except *MALRD1*, DUXAP8 and the other 9 PCGs were differentially expressed between tumor and non-tumor tissues (**Figure 2**). All PCGs and DUXAP8 were overexpressed in tumor tissues.

### Diagnosis, prognosis and joint-effect analysis of DUXAP8 and the top 10 PCGs

**Figure 3:**
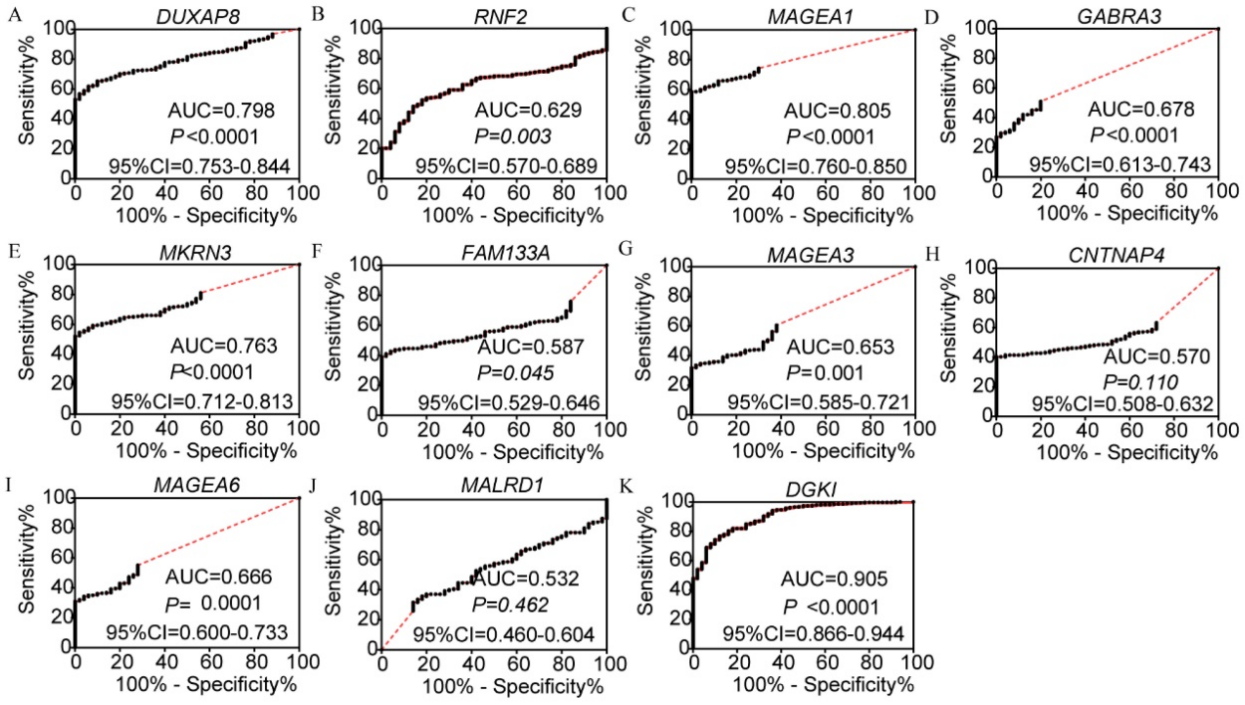
Diagnostic receiver operator curves of DUXAP8 and its co-expression-related protein-coding genes. A-K: Diagnostic receiver operator curves of DUXAP8, *RNF2*, *MAGEA1*, *GABRA3*, *MKRN3*, *FAM133A*, *MAGEA3*, *CNTNAP4*, *MAGEA6*, *MALRD1*, and *DGKI*, respectively.

**Figure 4:**
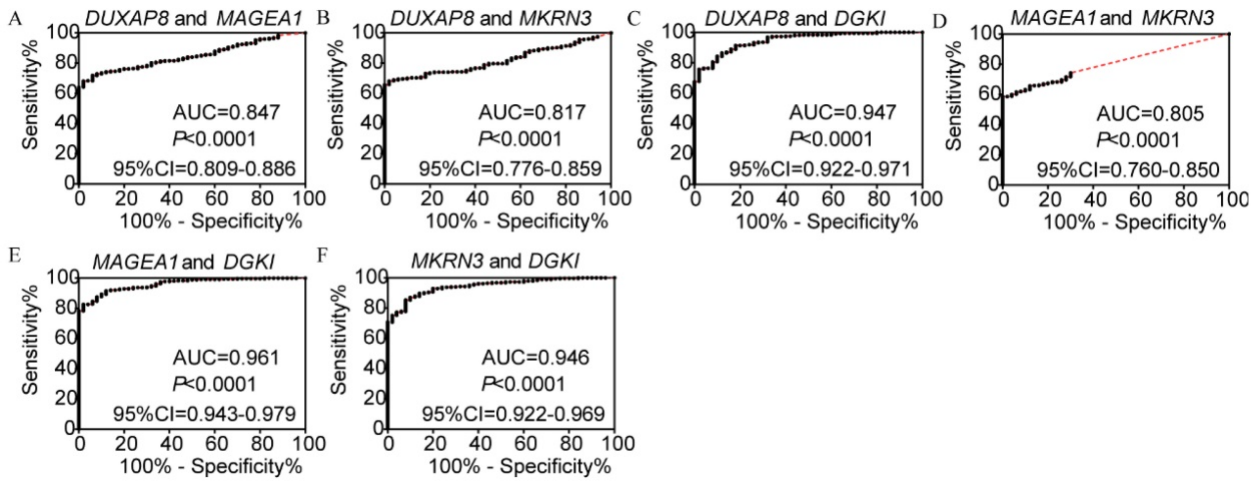
Joint-effect analysis of diagnostic receiver operator curves of DUXAP8 and diagnosis-related genes. A-F: Diagnostic receiver operator curves of DUXAP8 and *MAGEA1*; DUXAP8 and *MKRN3*; DUXAP8 and *DGKI*; *MAGEA1* and *MKRN3*; *MAGEA1* and *DGKI*; and *MKRN3* and *DGKI*, respectively.

In the diagnosis analysis, DUXAP8, *MEGEA1*, *MKRN3*, and *DGKI* were found to be potential diagnostic markers (AUC=0.798, 0.805, 0.763 and 0.905, respectively; p<0.0001, <0.0001, <0.0001, =0.0001 and <0.0001, respectively, (**Figure 3**). Other PCGs showed no diagnostic significance (all AUC<0.7). The joint-effect analysis of DUXAP8, *MEGEA1*, *MKRN3*, and *DGKI* indicated that their joint effect provided better diagnostic significance than when applied individually (AUC=0.847, 0.817, 0.947, 0.961 and 0.946, respectively; all p<0.0001; **Figure 4**), except for *MAGEA1* and *MKRN3*. The AUC value of this combination was equal to that of* MAGEA1* alone (both AUC=0.805, 95%CI=0.760-0.850;** Figure 3C, 4D**).

**Figure 5:**
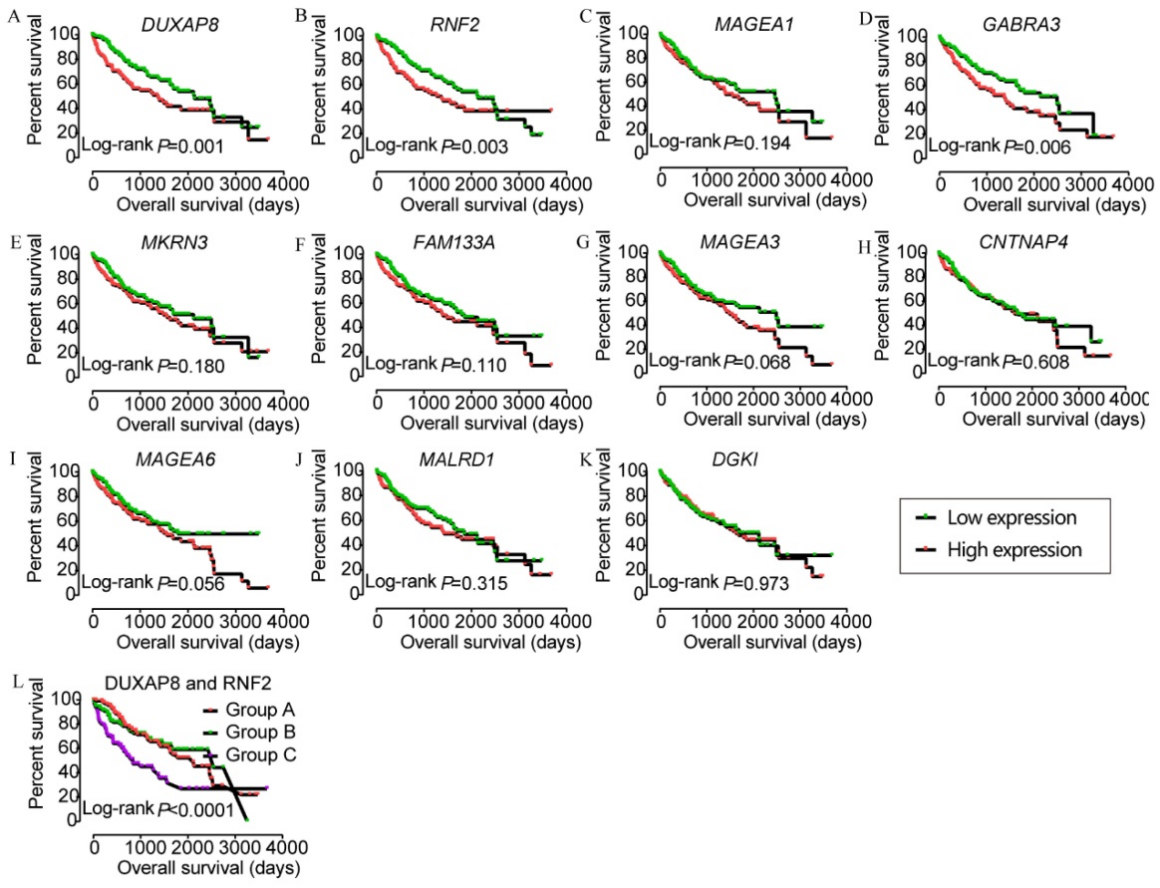
Kaplan-Meier plots of DUXAP8 and its co-expression-related protein-coding genes and joint-effect analysis of DUXAP8 and *RNF2*. A-L: Kaplan-Meier plots of DUXAP8, *RNF2*, *MAGEA1*, *GABRA3*, *MKRN3*, *FAM133A*, *MAGEA3*, *CNTNAP4*, *MAGEA6*, *MALRD1*, *DGKI*, and joint-effect analysis plot of DUXAP8 and *RNF2*, respectively.

In the prognostic analysis, *DUXAP8*, *RNF2*, and *GABRA3* showed prognostic value in univariate analysis (crude p=0.001, 0.003, and 0.006, respectively; **Figure 5**, **Table 2**). However, only *DUXAP8* and *RNF2* showed prognostic value in multivariate analysis, after adjustment for tumor stage, HBV infection, and radical resection factors (adjusted p=0.014 and 0.008, respectively;** Table 2**). Also, combination of DUXAP8 and RNF2 showed better prognostic value than individual effects (crude p<0.0001; adjusted p=0.001; **Figure 5L**, **Table 3**).

### Construction of risk score model and nomogram

**Figure 6:**
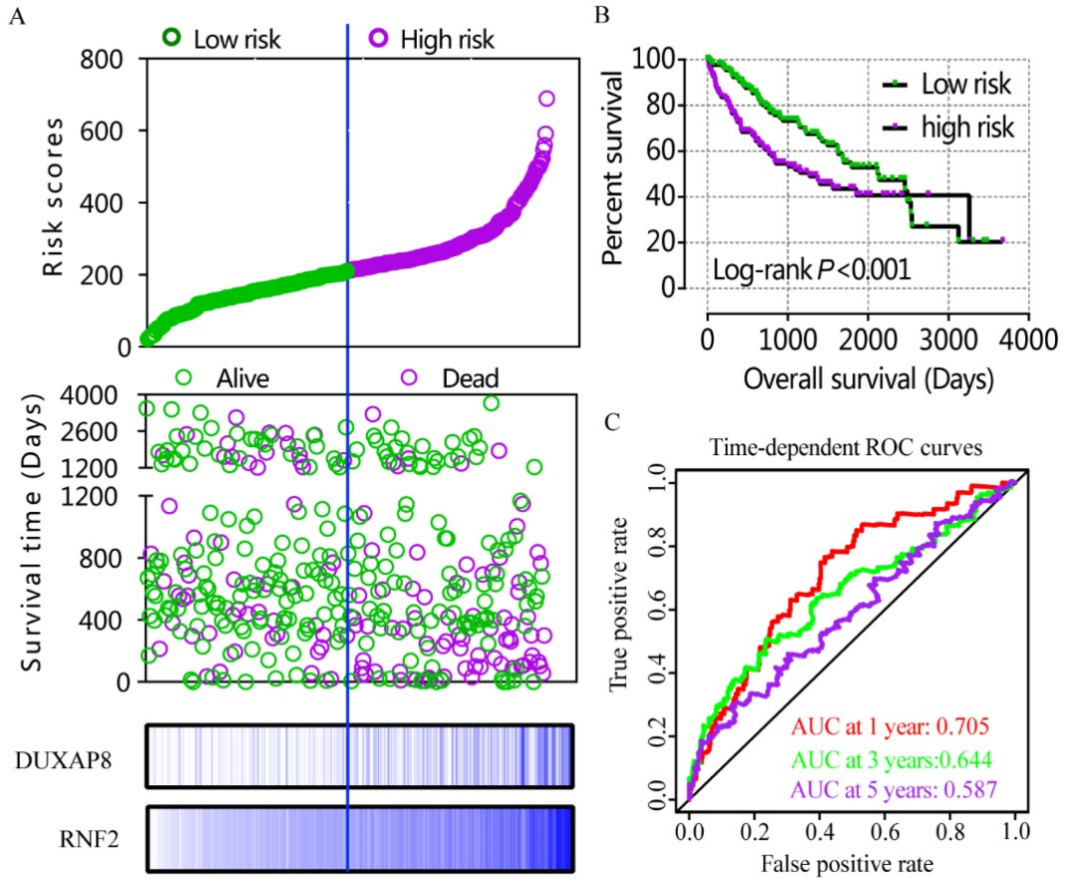
Risk score model, Kaplan-Meier plot, and time-dependent ROC curves. A: risk score model constructed using risk scores, patient survival status, DUXAP8 and *RNF2* expression heat maps; B: Kaplan-Meier plot of low and high risk groups; C: Time-dependent ROC curves of 1, 3, and 5year OS.

**Figure 7:**
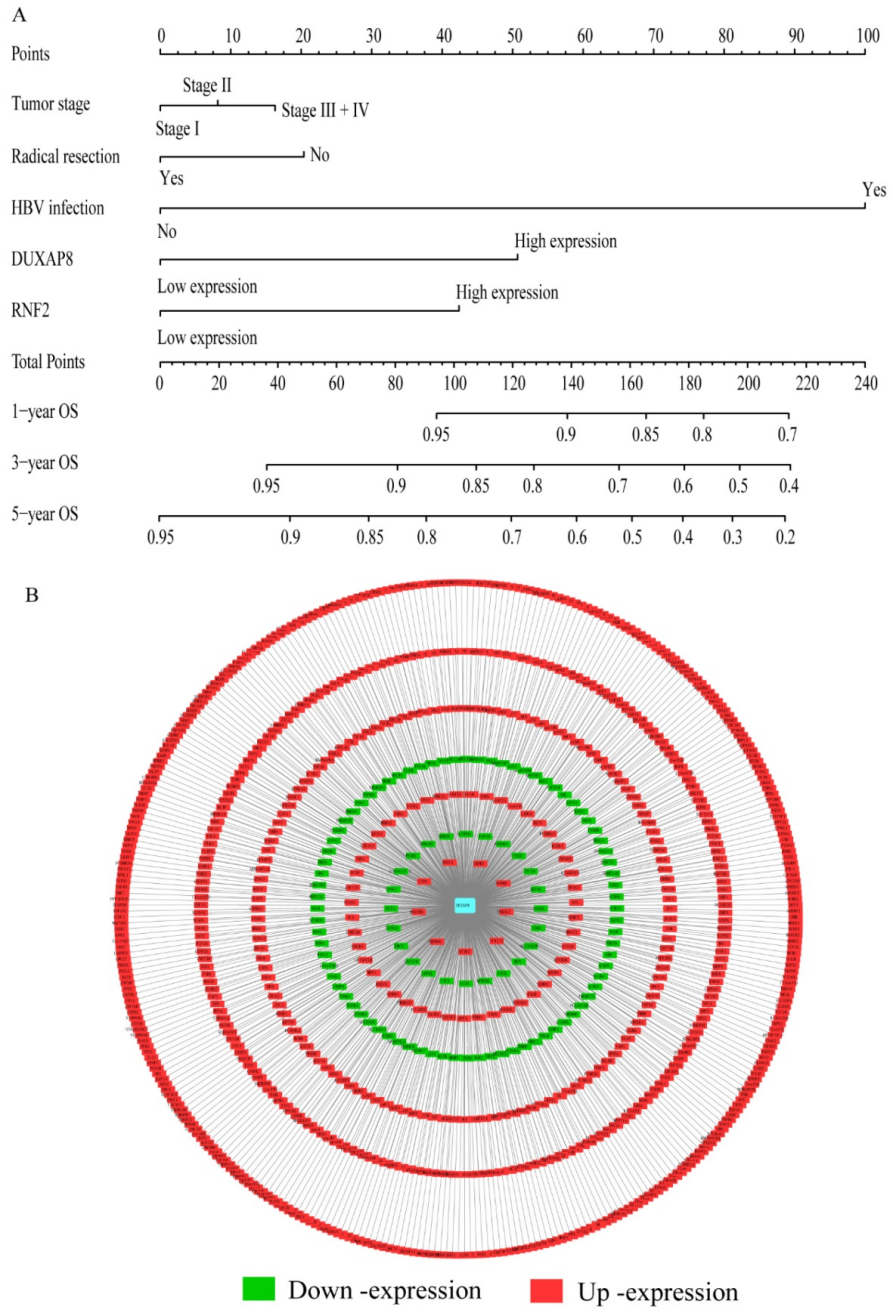
Nomogram and co-expression network of DUXAP8 and co-expression-related protein-coding genes. A: Nomogram constructed using DUXAP8, *RNF2*, tumor stage, radical resection, and hepatitis B virus infection status; B: Co-expression network between DUXAP8 and the co-expression-related protein-coding genes.

A risk score model was constructed using risk scores, patient survival status, and expressions levels DUXAP8 and *RNF2* (**Figure 6A**, **Table 4**). low DUXAP8 and *RNF2* expressions were associated with low risk and hence better prognosis (**Figure 6A-B**, **Table 5**). Time-dependent ROC curves suggested that the risk score model could predict the 1 and 3 years survival rate (AUC=0.705 and 0.644, respectively; **Figure 6C**). A nomogram was constructed using tumor stage, HBV infection status, radical resection status, and expressions of DUXAP8 and *RNF2* (**Figure 7A**). Low expressions of DUXAP8 and *RNF2*, tumor stage I, without HBV infection and radical resection, were correlated with low-risk scores, and hence better patient survival.

### Assessment of molecular mechanism using GSEA

**Figure 8:**
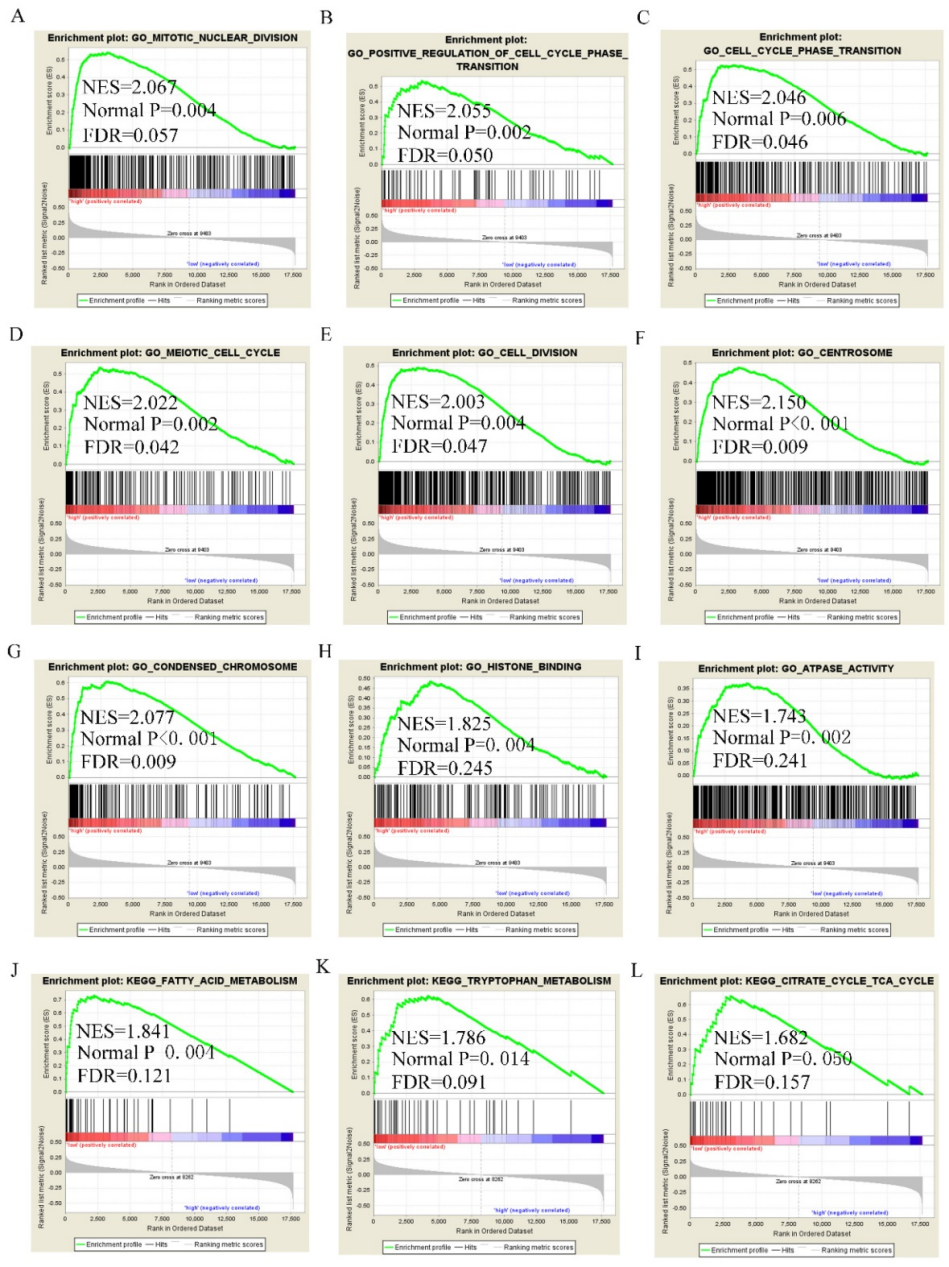
Gene set enrichment analysis of DUXAP8 of gene ontologies and KEGG pathways. A-I: Gene ontology results of DUXAP8; J-L: KEGG pathway results of DUXAP8.

**Figure 9:**
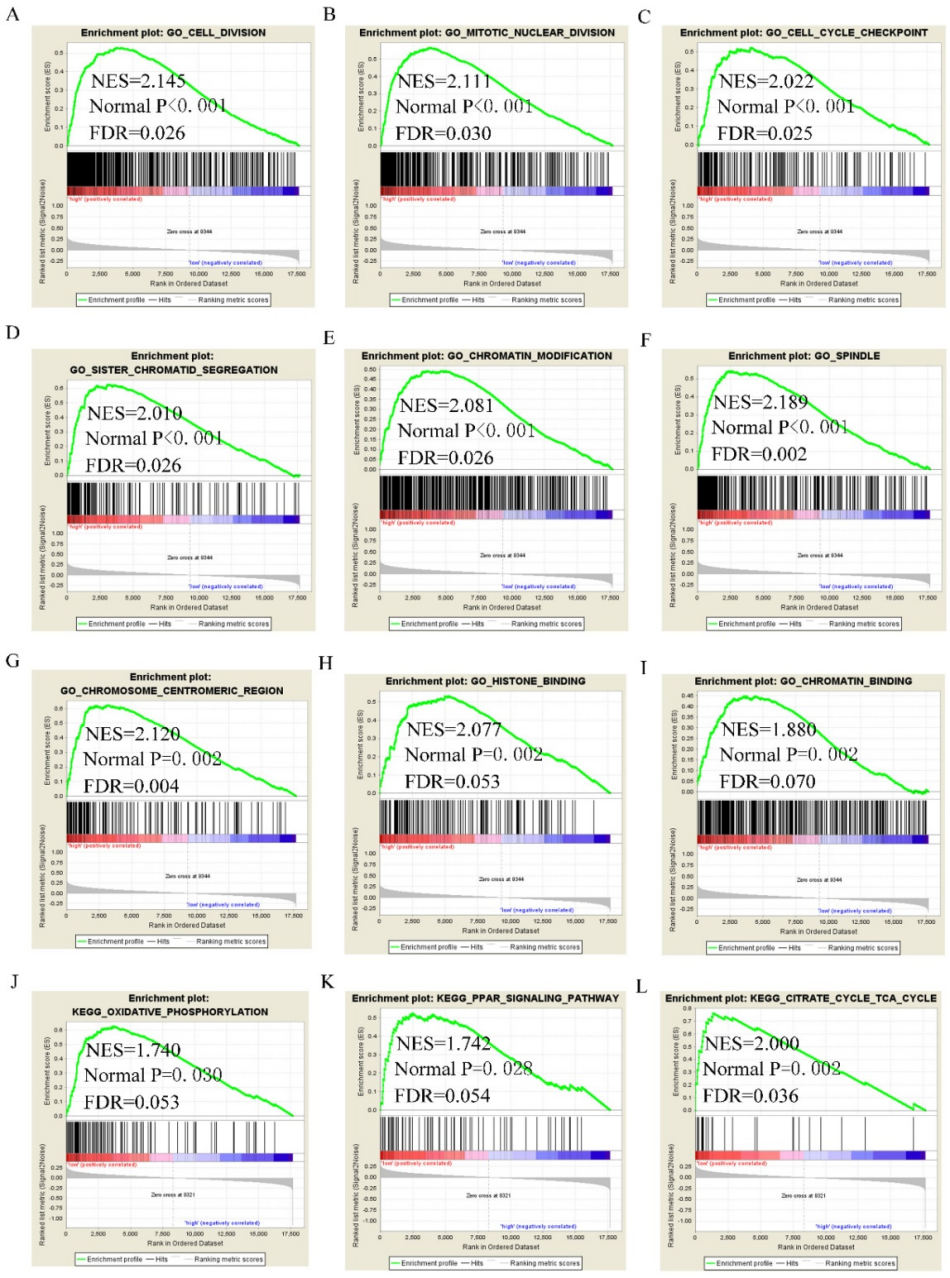
Gene set enrichment analysis of *RNF2* of gene ontologies and KEGG pathways. A-I: Gene ontology results of *RNF2*; J-L: KEGG pathway results of *RNF2*.

GSEA analysis of GO terms for DUXAP8 revealed that it was enriched in mitotic nuclear division, cell cycle phase transition, positive regulation of cell cycle phase transition, meiotic cell cycle, cell division, centrosome, chromosome condensation, and histone binding (**Figure 8A-I**). KEGG pathway analysis revealed that DUXAP8 was associated with fatty acid metabolism, tryptophan metabolism, and the citrate cycle (TCA cycle) pathways (**Figure 8J-L**). For *RNF2,* GO analysis revealed that it enriched in cell division, mitotic nuclear division, cell cycle checkpoint, sister chromatid segregation, chromatin modification and histone binding (**Figure 9A-I**). KEGG pathways analysis showed that *RNF2* was associated with oxidative phosphorylation, PPAR signaling pathway, and the TCA cycle pathways (**Figure 9J-L**).

### Construction of co-expression matrix, GGI and PPI network

**Figure 10:**
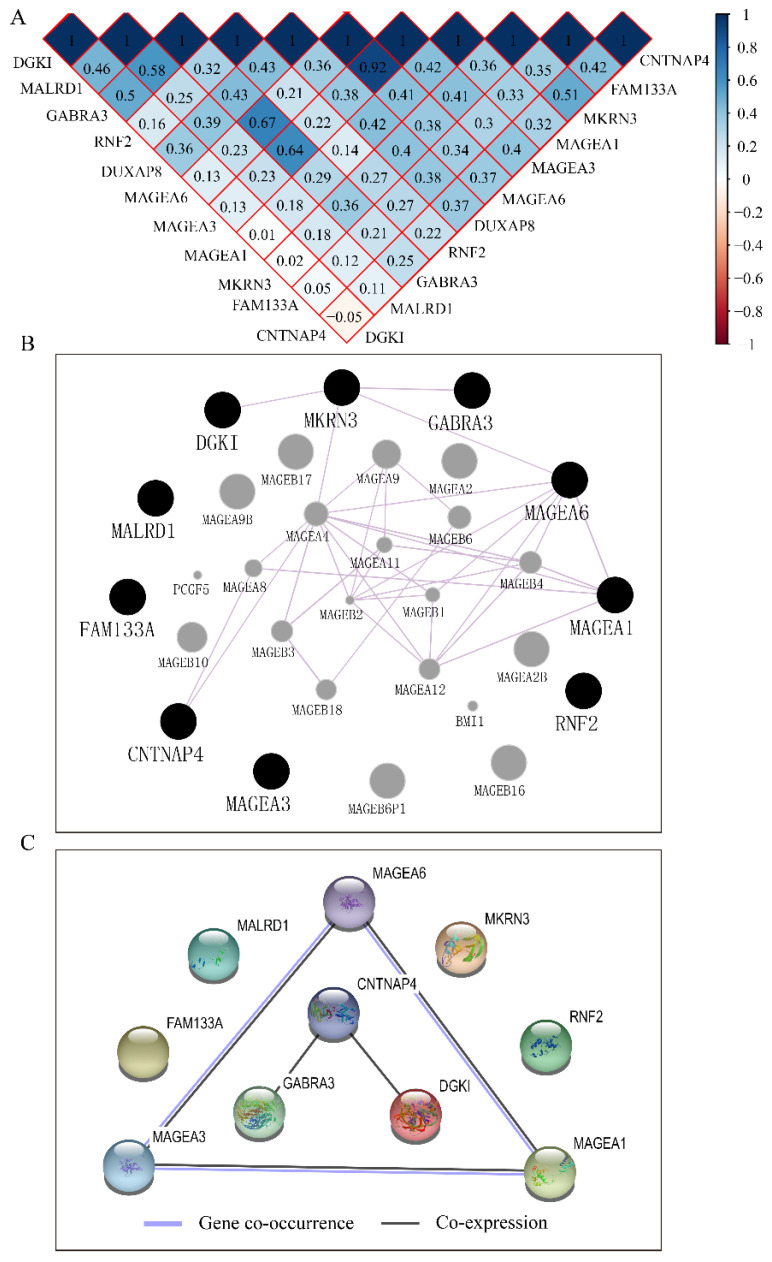
Co-expression matrix and gene-gene interaction and protein-protein interaction networks of LINC00668 and co-expression-related protein-coding genes. A: Co-expression matrix among DUXAP8, *RNF2*, *MAGEA1*, *GABRA3*, *MKRN3*, *FAM133A*, *MAGEA3*, *CNTNAP4*, *MAGEA6*, *MALRD1*, and *DGKI*; B: Co-expression network of gene-gene interactions among protein-coding genes; C: Protein-protein interaction network among *RNF2*, *MAGEA1*, *GABRA3*, *MKRN3*, *FAM133A*, *MAGEA3*, *CNTNAP4*, *MAGEA6*, *MALRD1*, and *DGKI*.

The co-expression matrix of DUXAP8 and PCGs revealed that most of them were positively correlated with each other. In the matrix, deep blue color indicated highly and positively correlated interactions (**Figure 10A**). The GGI network indicated that *CNTNAP4, MKRN3, DGKI, GABRA3, MAGEA6,* and *MAGEA1* were co-expressed with other genes (**Figure 10B**). Many *MAGE* family members interacted with them. Proteins encoded by *CNTNAP4, GABRA3, DGKI, MAGEA6, MAGEA3* and *MAGEA1* genes show co-expressional and co-occurrence relationship (**Figure 10C**).

### Identification of pharmacological targets

**Figure 11:**
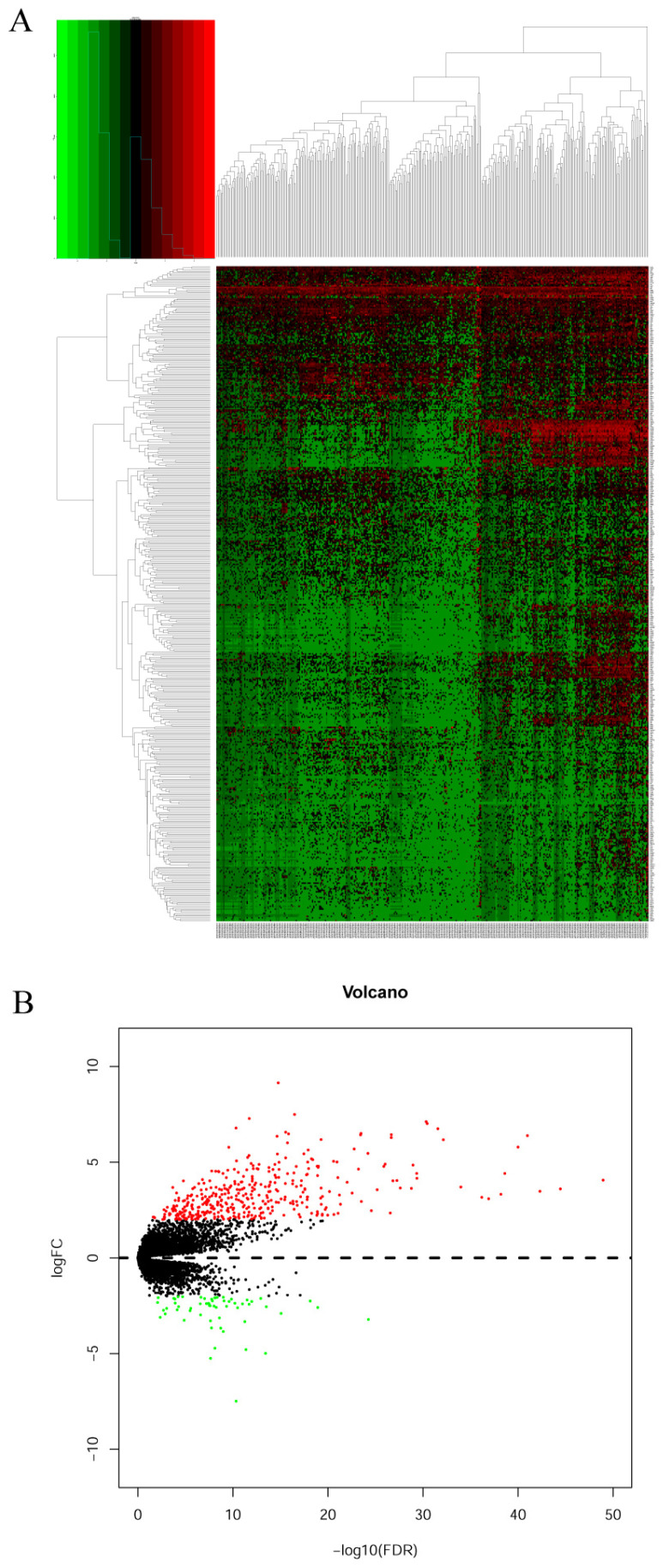
Heatmap and volcano plot of differentially expressed genes of DUXAP8. A: Heatmap of differentially expressed genes of DUXAP8; B: Volcano plot of differentially expressed genes of DUXAP8.

**Figure 12:**
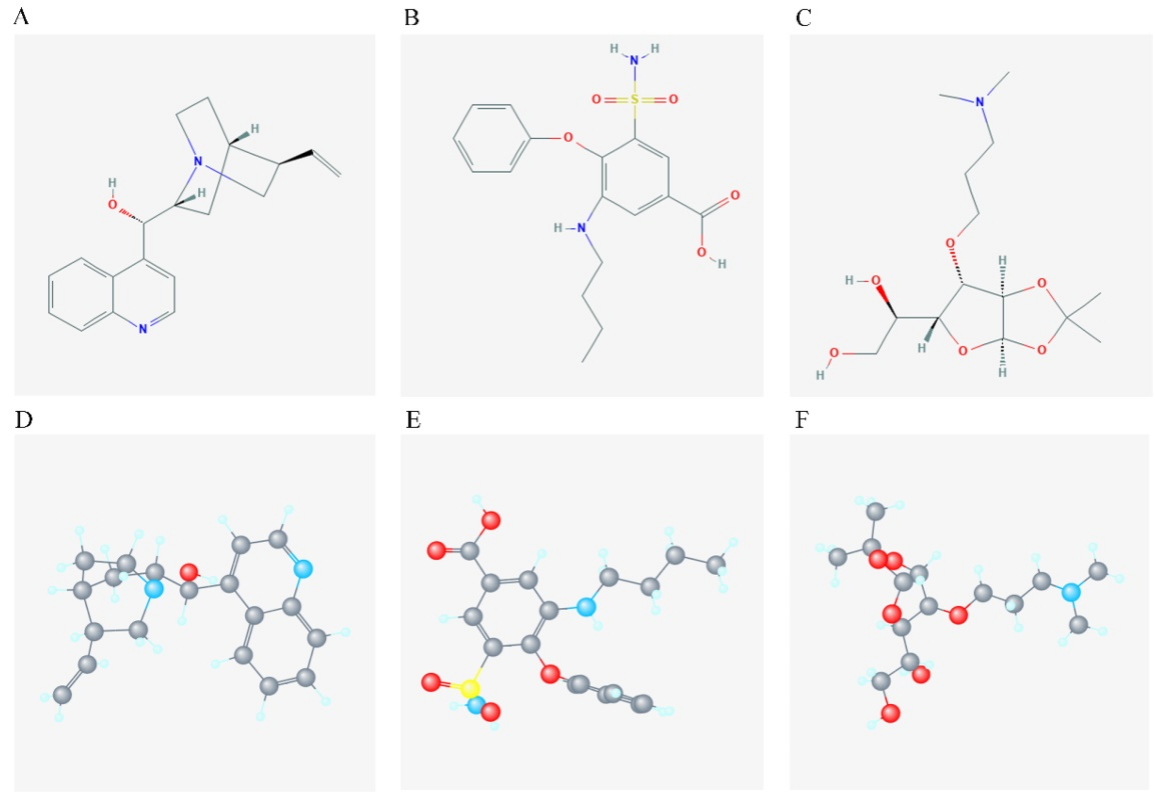
2D and 3D structure of the chemical compound of the 3 target drugs. A-C: 2D structure of cinchonine, bumetanide and amiprilose, respectively; D-F: 3D structure of cinchonine, bumetanide and amiprilose, respectively.

**Figure 13:**
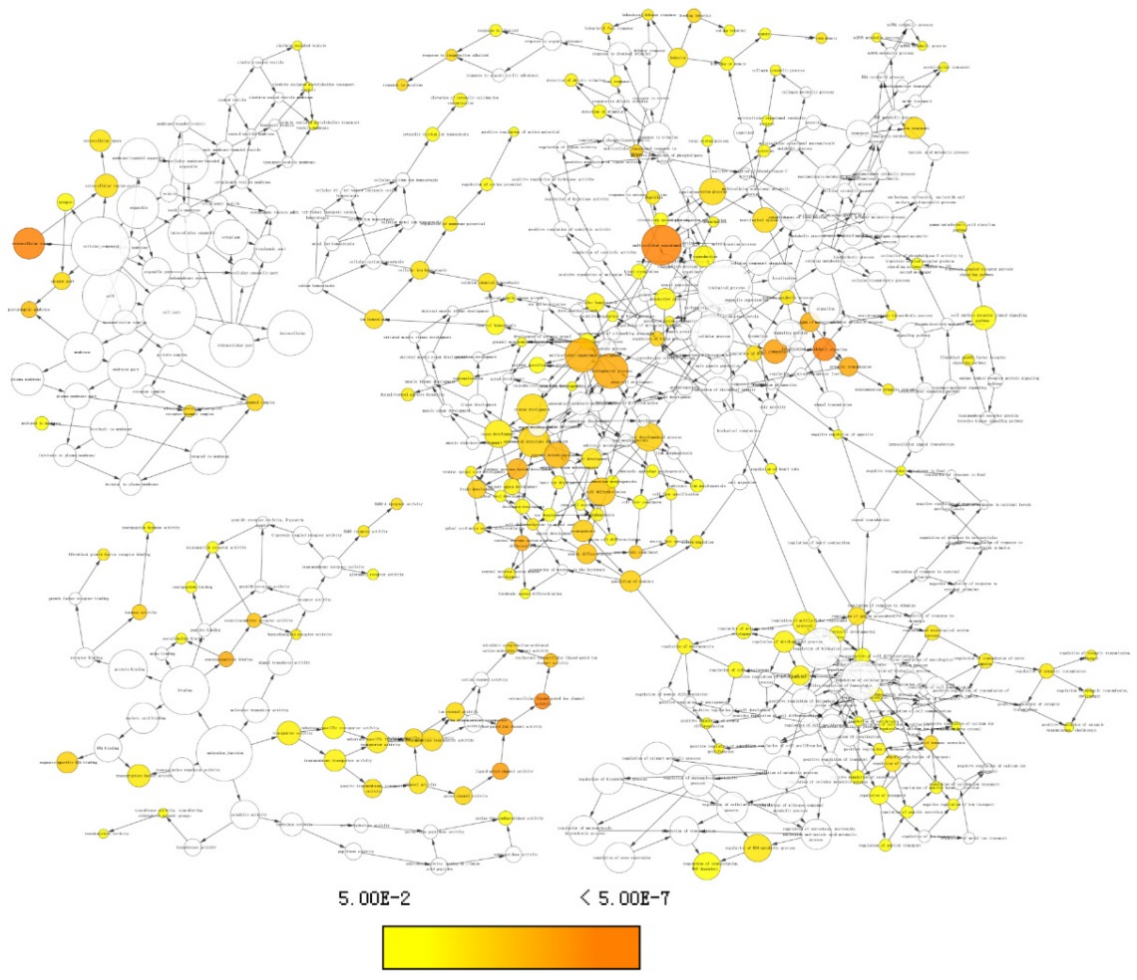
Enriched gene ontology terms network using differentially expressed genes.

DEGs were identified using edgeR and presented in heatmap and volcano plots as shown in **Figure 11**. Potential drugs targeting the DEGs were: cinchonine, bumetanide and amiprilose (**Table 6**). Detailed drug information is provided in **Supplementary Table 3**. The 2D and 3D structures of these three chemical compounds are in **Figure 12**. GO analysis was performed for the DEGs and results are shown in **Figure 13**. The DEGs were enriched in the synapse, extracellular region, postsynaptic membrane, ion channel complex, multicellular organismal development, and extracellular ligand-gated ion channel activity. The top 10 GO terms and KEGG pathways were: chemical synaptic transmission, ligand-gated ion channel activity, extracellular region, postsynaptic membrane, extracellular ligand-gated ion channel activity, neuroactive ligand-receptor interaction, and retrograde endocannabinoid signaling (**Table 7**). Detailed results of GO terms and KEGG pathways enrichment are shown in **Supplementary Tables 4 and 5**, respectively.

### Validations of potential significance of PCGs in public databases

Then, we further validated the potential significance of PCGs in HCC using HCCDB and oncomine databases. The diagnostic significance of MAGEA1, MKRN3, and DGKI was validated using oncomine database, which suggested diagnostic significance of *MAGEA1* (AUC=0.737, P=0.024, Supplementary Figure 1A, D) and *DGKI* (AUC=0.726, P=0.031, Supplementary Figure 1C, F), but not *MKRN* (AUC=0.655, P=0.058, Supplementary Figure 1B, E). The prognostic significance of *RNF2* was consistently validated in HCCDB, demonstrating the prognostic values of it in HCC (Log-rank P=0.001, 0.044, respectively, Supplementary Figure 1G-H).

## Discussion

The present study investigated the prognostic value of DUXAP8 and its PCGs. We found that DUXAP8, *RNF2*, *MAGEA1*, *GABRA3*, *MKRN3*, *FAM133A*, *MAGEA3*, *CNTNAP4*, *MAGEA6*, and *DGKI* are differentially expressed in liver tissues and are overexpressed in tumor tissues. Diagnostic analysis indicated that DUXAP8, *MEGEA1*, *MKRN3*, and *DGKI* are potential diagnostic biomarkers of HCC while DUXAP8 and *RNF2* are potential prognostic biomarkers of HCC. The joint-effect analysis of DUXAP8 and the PCGs showed better diagnostic and prognostic value compared to individual application. The constructed risk score model and nomogram showed good prediction for HCC prognosis. Molecular mechanism analyses revealed that DUXAP8 and *RNF2* are involved in mitotic nuclear division, cell division, regulation of cell cycle, phase transition, histone binding, oxidative phosphorylation, PPAR signaling pathway, and the TCA cycle. In addition, three target drugs; cinchonine, bumetanide, and amiprilose, were identified as targets of DUXAP8 using the Connectivity Map database. They are therefore likely to be ideal treatments for HCC treatment. Then, diagnostic and prognostic significance of PCGs were validated in HCCDB and oncomine databases.

Accumulating evidence indicates that non-coding RNAs regulate several physiological and pathological biological processes 40, 41. LncRNAs have been proposed to be potential diagnostic markers and therapeutic agents for various diseases 42. Dysregulation of lncRNAs may alter development of tumors 42. For instance, it is emerging that lncRNAs participate in tumorigenesis more actively than has been previously reported 42. Differential expression patterns of lncRNAs affect cell transformation, tumorigenesis, and metastasis 43. For instance, *H19, HOTAIR, MALAT1, TUG1, GAS5*, and *CCAT1*, were found to play important roles in tumor initiation and development 44-49. Numerous cancer-related lncRNAs modulates tumor invasion and metastasis processes 50-52. The transforming growth factor-β promotes the expression of ATB lncRNA in HCC cell lines, hence enhancing mesenchymal cell transition, cell invasion, and organ colonization of HCC cell lines 53.

DUXAP8, a 2107 bp RNA, was initially found to be overexpressed in gastric cancer (GC) tissues, its overexpression resulted in larger tumor size, advanced tumor stage, lymphatic metastasis, and poor prognosis of GC patients 54. It has been reported that DUXAP8 enhances GC cell proliferation and tumorigenesis, partly by epigenetically silencing PLEKHO1 expression through binding to PRC2, making it a potential biomarker for GC diagnosis and therapy 54. Hongzhi *et al.* found that DUXAP8 was overexpressed in pancreatic cancer (PC) tissues indicating poor OS in PC patients making it a potential therapeutic target 18. They also reported that DUXAP8 overexpression resulted in larger tumor size, advanced pathologic stage and poor OS of PC. This accelerates cell proliferation and tumorigenesis, partly by epigenetically silencing transcription of *CDKN1A* and *KLF2,* and by binding to *EZH2* and *LSD1* 18. Comprehensive profiling analysis revealed that DUXAP8 is upregulated in Bladder Cancer (BC). Its downregulation decreases cell growth, colony formation, invasion capacity and induces apoptosis of BC cells 20. Enrichment analysis of SNHG12 and DUXAP8 co-expressed PCGs found that they are involved in the cell cycle, focal adhesion, and PI3K-AKT signaling pathways 20. They also reported that DUXAP8 may regulate tumorigenesis and progression of BC 20.

Zheng *et al.* found that DUXAP8 is upregulated in renal cell carcinoma (RCC) tissues and promotes the proliferation and invasion of RCC cells by downregulating microRNA-126 expression 19. They also reported that DUXAP8 positively regulated RCC tumorigenesis and development 19. Elsewhere, DUXAP8 was found to be highly expressed in esophageal squamous cell cancer (ESCC) tissues and was associated with tumor stage, lymph node metastasis, and correlated with poor survival of ESCC patients 21. Functional experiments suggest that DUXAP8 modulates the occurrence of ESCC via the Wnt-β-catenin pathway. Specifically, it promotes cell proliferation, colony formation, and invasion of ESCC cells 21.

A genome-wide analysis revealed that DUXAP8 was highly expressed in esophageal cancer. GO enrichment analysis for DUXAP8 and its co-expressing PCGs showed that they were enriched in the cell cycle, cell division and DNA repair, suggesting an important role in the tumorigenesis and progression of esophageal cancer 24. It has been reported that knockdown of DUXAP8 resulted in clear cell cycle arrest in the G0/G1 phase ini non-small lung cancer cell lines, H1299, and H1975, which further decreased cyclin D1, CDK2, CDK4 and CDK6 expression in cell cycle process 23. Similarly, DUXAP8 was up-regulated in pancreatic cancer tissues. Knockdown of DUXAP8 expression arrested the cell cycle at the G0/G1 phase and induced apoptosis of pancreatic cancer cell lines 18. DUXAP8, SNHG12, and their PCGs were enriched in the cell cycle, focal adhesion, and PI3K-Akt signaling pathway 20. However, Hong-wei Ma's study using cell apoptosis and cell cycle regulation as factors reflecting cell growth of gastric cancer, revealed that the proportion of apoptotic cells were significantly decreased but not in the proportion in different phases 54.

We found cinchonine, bumetanide, and amiprilose as the potential drugs targeting DUXAP8 in HCC. Cinchonine (C_19_H_22_N_2_O) is a natural compound used as an antimalarial drug 55. It exerts antitumor effects with high activity and low toxicity 56. Moreover, cinchonine inhibited cell proliferation and promoted apoptosis by activating caspase-3 dependence in human liver cancer cells 57. Similarly, it was reported that cinchonine induced apoptosis of Hela and A549 cells by targeting TRAF6, suggesting that cinchonine has antitumor effects 58. Bumetanide is a commonly used diuretic drug in clinical practice. A previous study showed that bumetanide, a SLC12A1 antagonist, inhibited cell proliferation, tumorigenesis, and metastasis in HCC cell lines 59. The study also suggested that bumetanide slows down tumor growth by interfering with the cell cycle rather than by inducing cytotoxicity 59. Studies testing the ability of bumetanide to enhance tumor necrosis in a rat model of N1-S1 HCC. They found that bumetanide treatment increased tumor necrosis in N1-S1 HCC during transarterial embolization compared to during transarterial embolization alone 60. Amiprilose is a synthetic carbohydrate used for patients with rheumatoid arthritis. It has anti-inflammatory and immunomodulatory properties 61. None has reported whether it has any effects on liver cancer. Our findings showed that cinchonine, bumetanide, and amiprilose are candidate drugs that target DUXAP8 in HCC. However, the specific mechanisms of action of these drugs in HCC deserves further studies.

Our study indicates that higher expression of DUXAP8 correlated with poor prognosis in HCC. We also found that DUXAP8 is involved in the mitotic nuclear division, cell division, regulation of cell cycle phase transition, histone binding, oxidative phosphorylation, and the TCA cycle pathways. Therefore, we infer that DUXAP8 functions as an oncogene in HCC. This conclusion is in agreement with previous reports in ESCC, PC, BC, GC, and RCC.

Live cell imaging or readouts of active cellular processes have revealed that lncRNAs play various roles in cellular pathologies and can influence the efficacy of new therapeutic targets for cancer 62. Our study identified three potential drugs targeting DUXAP8 in HCC. Although several related studies focusing on DUXAP8 in HCC were previously reported 63-65, our study has several main findings that could distinguished from them, such as in both diagnostic and prognostic significance of DUXAP8 and it-related PCGs, construction of risk score model and nomogram, potential target drugs toward DXUAP8 aspects.

We show that* SNF2* is overexpressed in tumor tissues leading to poor patient survival. Molecular mechanism results demonstrate that *SNF2* is involved in cell division, mitotic nuclear division, cell cycle checkpoint, sister chromatid segregation, chromatin modification, histone binding, oxidative phosphorylation, PPAR signaling pathway, and the TCA cycle. Therefore, we speculate that SNF2 is an oncogene in HCC A recent study showed that downregulation of* SNF2*, a co-expression-related gene of DUXAP8, decreases cell growth and metastases of HCC cells. 66. The study showed that *RNF2* promoted HCC cell proliferation by accelerating cell cycle progression 66. Similar findings have been reported for *SNF2* in several malignancies^67^. In conclusion, our study is consistent with previous studies showing that *SNF2* is an oncogene in HCC.

This study for the first time demonstrates the oncogenic role, diagnostic and prognostic value of DUXAP8 in HCC. This study has some limitations that need to be recognized. First, our findings concerning DUXAP8 and its PCGs need to be validated using a larger population. Second, well-designed functional trials are necessary to identify deeper mechanisms of DUXAP8 and its PCG in HCC. Thus, further clinical trials are required to assess the translational potential of DUXAP8.

## Conclusions

This study investigated the functions of DUXAP8 and its PCGs in HCC. We found that DUXAP8 and its PCGs are differentially expressed in the liver and overexpressed in tumor tissues. Diagnostic and prognostic analysis indicated that DUXAP8, *MEGEA1*, *MKRN3*, and *DGKI* can be used to diagnosis HCC, while DUXAP8 and *RNF2* can be used to predict the prognosis of HCC. In addition, we found that DUXAP8 and its PCGs demonstrated better diagnostic and prognostic performance when these factors were used jointly as opposed to single application. A risk score model and nomogram exhibited good prognostic prediction performance of DUXAP8 and its PCGs in HCC. Molecular analyses revealed that DUXAP8 and PCGs are involved in mitotic nuclear division, cell division, regulation of cell cycle phase transition, oxidative phosphorylation, and PPAR signaling pathway. Furthermore, three potential target drugs; cinchonine, bumetanide, and amiprilose, which were identified as candidate drugs targeting DUXAP8 in HCC. This study for the first time demonstrates the oncogenic role, diagnostic and prognostic value of DUXAP8 in HCC. Thus, further clinical trials are required to assess the translational potential of DUXAP8.

## Supplementary Material

Supplementary figure.

Supplementary tables.

## Figures and Tables

**Table 1:** Top 10 protein-coding genes with DUXAP8

LncRNA	PCG	Correlation	95% CI	P value
DUXAP8	RNF2	0.43	0.35-0.51	1.90E-18
DUXAP8	MAGEA1	0.42	0.33-0.5	4.86E-17
DUXAP8	GABRA3	0.41	0.32-0.49	4.55E-16
DUXAP8	MKRN3	0.39	0.30-0.47	8.87E-15
DUXAP8	FAM133A	0.38	0.29-0.47	2.30E-14
DUXAP8	MAGEA3	0.38	0.29-0.46	4.70E-14
DUXAP8	CNTNAP4	0.36	0.27-0.44	1.38E-12
DUXAP8	MAGEA6	0.36	0.26-0.44	1.53E-12
DUXAP8	MALRD1	0.36	0.26-0.44	1.84E-12
DUXAP8	DGKI	0.35	0.26-0.44	3.35E-12

Note: PCG: protein-coding gene, CI: confidence interval.

**Table 2:** Prognostic analysis of *DUXAP8* and target genes for overall survival

Long non-coding RNA	Patients	Overall survival					
	(n=370)	No. of event	MST (days)	HR (95%CI)	Crude *P* value	HR (95%CI)	Adjusted* P* value^§^
*DUXAP8*							
Low expression	185	56	2131	Ref.	**0.001**	Ref.	**0.014**
High expression	185	74	1372	**1.768 (1.247-2.507)**		**1.632 (1.104-2.412)**	
*RNF2*							
Low expression	185	57	2131	Ref.	**0.003**	Ref.	**0.008**
High expression	185	73	1372	**1.704 (1.199-2.420)**		**1.700 (1.146-2.519)**	
*MAGEA1*							
Low expression	185	59	2456	Ref.	0.194	Ref.	0.480
High expression	185	71	1397	1.258 (0.889-1.780)		1.147 (0.784-1.678)	
*GABRA3*							
Low expression	185	55	2486	Ref.	**0.006**	Ref.	0.103
High expression	185	75	1372	**1.631 (1.149-2.313)**		1.378 (0.937-2.026)	
*MKRN3*							
Low expression	185	58	2116	Ref.	0.180	Ref.	0.672
High expression	185	72	1560	1.267 (0.896-1.792)		1.086 (0.740-1.595)	
*FAM133A*							
Low expression	185	61	1852	Ref.	0.110	Ref.	0.472
High expression	185	69	1397	1.325 (0.938-1.872)		1.152 (0.784-1.694)	
*MAGEA3*							
Low expression	185	57	2486	Ref.	0.068	Ref.	0.207
High expression	185	73	1490	1.381 (0.976-1.955)		1.280 (0.872-1.878)	
*CNTNAP4*							
Low expression	185	65	1694	Ref.	0.608	Ref.	0.952
High expression	185	65	1624	1.094 (0.776-1.544)		0.988 (0.675-1.446)	
*MAGEA6*							
Low expression	185	56	1791	Ref.	0.056	Ref.	0.186
High expression	185	74	1560	1.404 (0.992-1.988)		1.298 (0.882-1.909)	
*MALRD1*							
Low expression	185	61	1852	Ref.	0.315	Ref.	0.707
High expression	185	69	1423	1.194 (0.845-1.686)		1.076 (0.734-1.577)	
*DGKI*							
Low expression	185	61	2116	Ref.	0.973	Ref.	0.170
High expression	185	69	1624	1.006 (0.712-1.421)		0.763 (0.518-1.123)	

**Note: §**: P values were adjusted for radical resection, tumor stage and HBV infection; Bold indicates significant P values, NA: not available; MST: median survival time; HR: hazard ratio; 95%CI: 95% confidence interval.

**Table 3:** Joint-effect analysis of *DUXAP8* and *RNF2* for overall survival

Group	*DUXAP8* expression	*RNF2*	Overall survival					
			Events/total	MST (Days)	Crude HR (95%CI)	Crude *P* value	Adjusted HR (95%CI)	Adjusted *P* value^ɸ^
A	Low	Low	39/120	2116	Ref.	**<0.0001**	Ref.	**0.001**
B	Low	High	35/130	2542	1.001 (0.633-1.584)	0.995	1.030 (0.626-1.694)	0.908
	High	Low						
C	High	High	56/120	837	2.268 (1.498-3.433)	<0.001	2.184 (1.364-3.497)	0.001

**Note:** ɸ: P values were adjusted for radical resection, tumor stage and HBV infection; Bold indicates significant P values, NA: not available; MST: median survival time; HR: hazard ratio; 95%CI: 95% confidence interval.

**Table 4:** Construction of risk score model

Variables	β	SE	Wald	*P* value	HR (95% CI)
Tumor stage I			11.611	0.003	
Stage II	0.153	0.268	0.328	0.567	1.166 (0.690-1.970)
Stage III + IV	0.734	0.224	10.683	0.001	2.083(1.341-3.233)
Radical resection	0.068	0.356	0.037	0.848	1.071(0.533-2.152)
HBV infection	-0.896	0.264	11.545	0.001	0.408(0.244-0.685)
*DUAXP8*	0.382	0.206	3.432	0.064	1.465(0.978-2.195)
*RNF2*	0.433	0.207	4.366	0.037	1.542(1.027-2.315)

Note: SE: standard error, HR: hazard ratio, CI: confidence interval.

**Table 5:** Overall survival analysis of risk score model

Group	Overall survival					
	Events/total	MST (Days)	Crude HR (95%CI)	Crude *P* value	Adjusted HR (95%CI)	Adjusted *P* value^ɸ^
Risk score model						
Low risk	54/185	2131	Ref.	**0.001**	Ref.	**0.010**
High risk	76/185	1271	**1.808 (1.274-2.565)**		**1.672 (1.130-2.477)**	

**Note:** ɸ: P values were adjusted for radical resection, tumor stage and HBV infection; Bold indicates significant P values. Abbreviations: NA: not available; MST: median survival time; HR: hazard ratio; 95%CI: 95% confidence interval.

**Table 6:** Top 7 pharmacological target and drugs of DUXAP8

Name	PubChem CID	Mean	Enrichment	P value
D-Cinchonine	90454	-0.411	-0.755	0.00734
Bumetanide	2471	-0.407	-0.708	0.01486
Scopolamine	300322	0.383	0.694	0.01842
Trolox C	40634	0.164	0.690	0.01920
Harmaline	3564	0.430	0.660	0.03083
Cefotetan	53025	0.504	0.736	0.03609
Amiprilose	9798098	-0.413	-0.641	0.03981

**Table 7:** Top 10 gene ontologies and KEGG pathways of differentially expressed genes

Category	Term	Count	%	P value
BP	GO: 0007268~chemical synaptic transmission	19	4.241071429	3.86E-06
MF	GO: 0015276~ligand-gated ion channel activity	8	1.785714286	6.26E-06
CC	GO: 0005576~extracellular region	62	13.83928571	9.28E-06
BP	GO: 0035094~response to nicotine	8	1.785714286	1.08E-05
CC	GO: 0045211~postsynaptic membrane	17	3.794642857	1.40E-05
CC	GO: 0005615~extracellular space	53	11.83035714	2.78E-05
MF	GO: 0005230~extracellular ligand-gated ion channel activity	7	1.5625	4.40E-05
BP	GO: 0007586~digestion	9	2.008928571	5.29E-05
MF	GO: 0005179~hormone activity	10	2.232142857	1.13E-04
BP	GO: 0007271~synaptic transmission, cholinergic	7	1.5625	1.16E-04
KEGG pathway	hsa04080: Neuroactive ligand-receptor interaction	30	6.696428571	1.09E-14
KEGG pathway	hsa04972: Pancreatic secretion	10	2.232142857	5.19E-05
KEGG pathway	hsa04723: Retrograde endocannabinoid signaling	9	2.008928571	5.48E-04
KEGG pathway	hsa05033: Nicotine addiction	6	1.339285714	8.05E-04
KEGG pathway	hsa04727: GABAergic synapse	8	1.785714286	9.70E-04
KEGG pathway	hsa04974: Protein digestion and absorption	8	1.785714286	0.001191854
KEGG pathway	hsa05032: Morphine addiction	7	1.5625	0.006772348
KEGG pathway	hsa05218: Melanoma	5	1.116071429	0.042731956
KEGG pathway	hsa04725: Cholinergic synapse	6	1.339285714	0.055498778
KEGG pathway	hsa00053: Ascorbate and aldarate metabolism	3	0.669642857	0.089180199

Note: KEGG, Kyoto Encyclopedia of Genes and Genomes, BP: biological process, CC: cellular component, MF: molecular function, FDR: false discovery rate.
