# Supplementary material for: Clinical significance of long non-coding RNA DUXAP8 and its protein coding genes in hepatocellular carcinoma
Source: J Cancer. 2020 Aug 25;11(20):6140–56. doi: 10.7150/jca.47902 (PMC7477403; doi:10.7150/jca.47902)
Supplement: Supplementary file 1 — Supplementary figure. [file jcav11p6140s1.pdf]

Supplementary figure legends

Supplementary figure 1. Validation of diagnostic and prognostic significance of DUXAP8-related protein-coding genes. A-C: Differential expressions of *MAGEA1*, *MKRN3*, and *DGKI*; D-F: Diagnostic receiver operating characteristic curves of *MAGEA1*, *MKRN3*, and *DGKI*; G-H: Survival plots of RNF2 in HCC in HCCDB15 and HCCDB18 datasets.

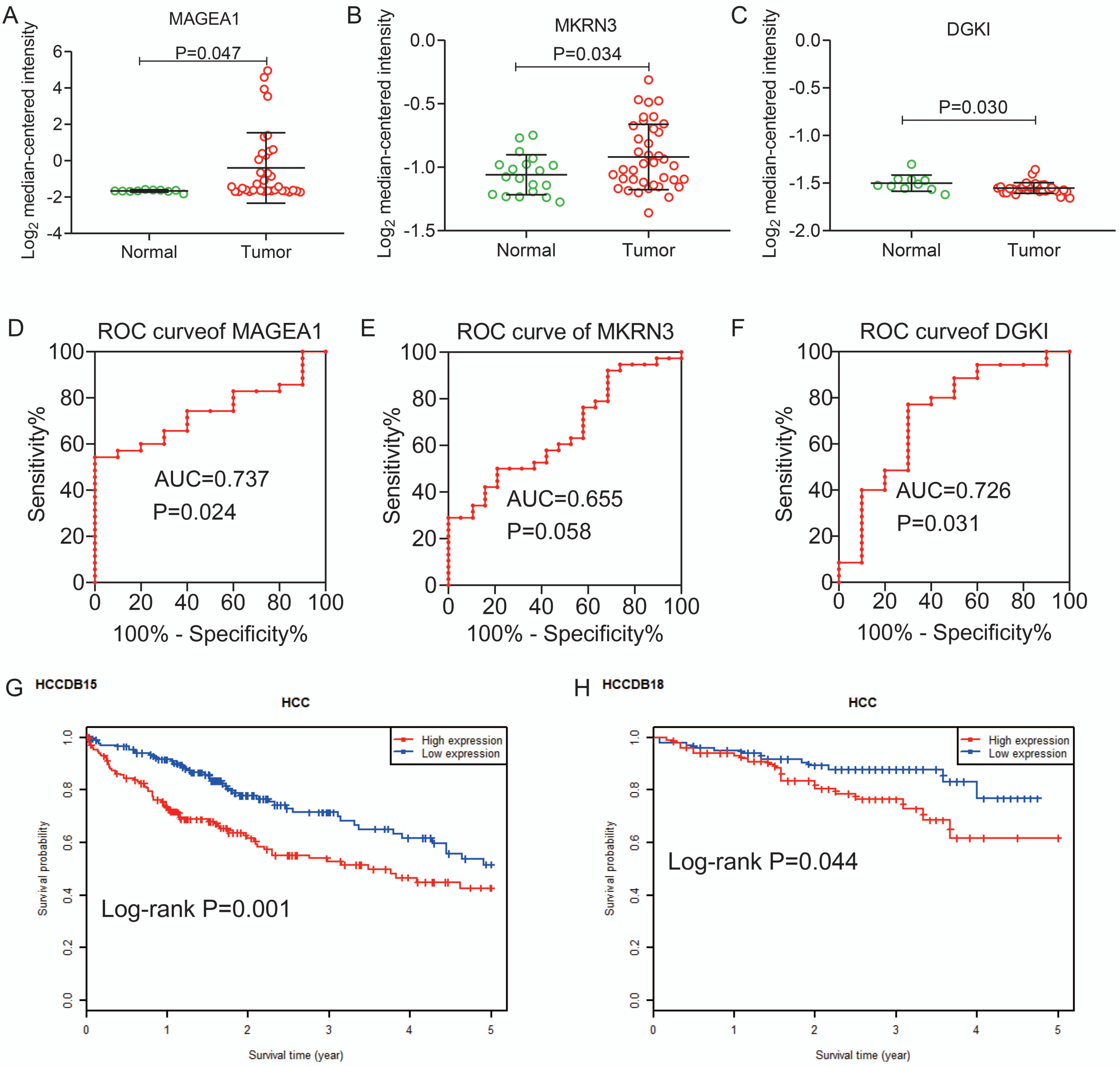

A
MAGEA1
P=0.047
Log2 median-centered intensity
Normal
Tumor
B
MKRN3
P=0.034
Log2 median-centered intensity
Normal
Tumor
C
DGKI
P=0.030
Log2 median-centered intensity
Normal
Tumor
D
ROC curveof MAGEA1
AUC=0.737
P=0.024
Sensitivity%
100% - Specificity%
E
ROC curve of MKRN3
AUC=0.655
P=0.058
Sensitivity%
100% - Specificity%
F
ROC curveof DGKI
AUC=0.726
P=0.031
Sensitivity%
100% - Specificity%
G
HCCDB15
HCC
High expression
Low expression
Log-rank P=0.001
Survival probability
Survival time (year)
H
HCCDB18
HCC
High expression
Low expression
Log-rank P=0.044
Survival probability
Survival time (year)
